# A rapid assessment of wastewater for genomic surveillance of SARS-CoV-2 variants at sewershed scale in Louisville, KY

**DOI:** 10.1101/2021.03.18.21253604

**Published:** 2021-03-26

**Authors:** J. L. Fuqua, E.C. Rouchka, S. Waigel, K. Sokoloski, D. Chung, W. Zacharias, M. Zhang, J. Chariker, D. Talley, I. Santisteban, A. Varsani, S. Moyer, R. H. Holm, R. A. Yeager, T. Smith, A. Bhatnagar

**Affiliations:** 1 Department of Pharmacology and Toxicology, University of Louisville, 505 S. Hancock St., Louisville, KY 40202, United States; 2 Center for Predictive Medicine, University of Louisville, 505 S. Hancock St., Louisville, KY 40202, United States; 3 Department of Computer Science and Engineering, University of Louisville, 522 East Gray St., Louisville, KY 40202, United States; 4 KY-INBRE Bioinformatics Core, University of Louisville, 522 East Gray St., Louisville, KY 40202, United States; 5 Department of Medicine, University of Louisville, 530 S. Hancock Jackson St., Louisville, KY 40402, United States; 6 Department of Microbiology and Immunology, University of Louisville, 505 S. Hancock St., Louisville, KY 40202, United States; 7 Department of Neuroscience Training, University of Louisville, 505 S. Hancock St, Louisville, KY 40202; 8 Louisville/Jefferson County Metropolitan Sewer District, Morris Forman Water Quality Treatment Center, 4522 Algonquin Parkway, Louisville KY 40211, United States; 9 The Biodesign Center of Fundamental and Applied Microbiomics, School of Life Sciences, Center for Evolution and Medicine, Arizona State University, Tempe, AZ 85287, United States; 10 Department of Health Management and System Sciences, School of Public Health and Information Sciences, University of Louisville, 485 E. Gray St., Louisville, KY 40202, United States; 11 Department of Public Health and Wellness, Louisville Metro Government, 400 E. Grays St., Louisville, KY 40202, United States; 12 Christina Lee Brown Envirome Institute, University of Louisville, 302 E. Muhammad Ali Blvd., Louisville, KY 40202, United States; 13 Department of Environmental and Occupational Health Sciences, School of Public Health and Information Sciences, University of Louisville, 485 E. Gray St., Louisville, KY 40202, United States

## Abstract

In this communication, we report on the genomic surveillance of SARS-CoV-2 using wastewater samples in Jefferson County, KY. In February 2021, we analyzed seven wastewater samples for SARS-CoV-2 genomic surveillance. Variants observed in smaller catchment areas, such as neighborhood manhole locations, were not necessarily consistent when compared to associated variant results in downstream treatment plants, suggesting catchment size or population could impact the ability to detect diversity.

The successful viral detection of severe acute respiratory syndrome coronavirus 2 (SARS-CoV-2) RNA in wastewater at various pooled scales (1–4) and discovery in the USA of B.1.1.7, B.1.351 and P.1 variants (5), has led to an interest in developing reliable population-level wastewater viral genomic surveillance.

The diversity of SARS-CoV-2 sequences reported to be circulating in the USA, have been determined by sequencing clinical samples; however, these variants can also be surveilled by sequencing wastewater samples (6–9). As of March 2021, the variants of concern - B.1.1.7, B.1.351, and P.1 have been widely detected in clinical samples from 47 states in the USA. In Kentucky, only five clinical cases have been linked to the presence of these variants (5), which could indicate incomplete surveillance. Broadening the application of genomic surveillance using wastewater in the community could enhance SARS-CoV-2 variant population monitoring.

In this communication, we report on the genomic surveillance of SARS-CoV-2 using wastewater samples in Jefferson County, KY. Samples were collected from manholes and treatment facilities, covering populations of 8,000 to 350,000 people (Table 1). RNA isolated from wastewater samples was used to quantify SARS-CoV-2 and analyze the genetic variation through high-throughput sequencing (See Supplementary Methods). Bioinformatics approaches were used to rapidly identify single nucleotide genetic alterations, which were compared with known variants of interest and concern.

In February 2021, we analyzed seven wastewater samples for SARS-CoV-2 genomic surveillance (Figure 1). We did not detect genetic variations indicative of any current variant of concern, beyond the widespread D614G spike protein mutation (Supplementary Methods Tables 2–5). In all samples, we identified at least four of ten mutations consistent with the presence of the variant of interest B.1.429, and one sample contained seven of ten mutations (Table 2). The B.1.429 variant was confirmed in patient samples in Kentucky in January 2021 (10), and a single patient in the study area was reported to be positive for B.1.1.7 on February 9, 2021 (11). With our current metrics we flagged sites 833, 891, and Treatment plant #2 for potential presence of variant B.1.429 (3/7 sites). Differences in the scale of sample pooling in the community revealed unanticipated inconsistencies in variant representation. Specifically, variants observed in smaller catchment areas, such as neighborhood manhole locations, were not observed in downstream treatment plants, suggesting catchment size or population could impact the ability to detect diversity.

Given the highly variable viral genome sequence coverage recovered from wastewater samples, there is an urgent need to develop a set of consistent thresholds constituting positive/negative presence of a variant. Monitoring SARS-CoV-2 variants in wastewater may warn of an emerging variant of concern and identify variant dominance occurring when a new variant is introduced in a community. Wastewater genetic monitoring may be particularly useful in the context of limited clinical sample sequencing capacity because a broad perspective on the genetic diversity can be obtained from a few samples. To develop comprehensive epidemiological frameworks required to guide policy, population-level wastewater surveillance of viral genetic diversity should be complemented by clinical sample testing.

## Supplementary Material

1

## Figures and Tables

**Figure 1.**
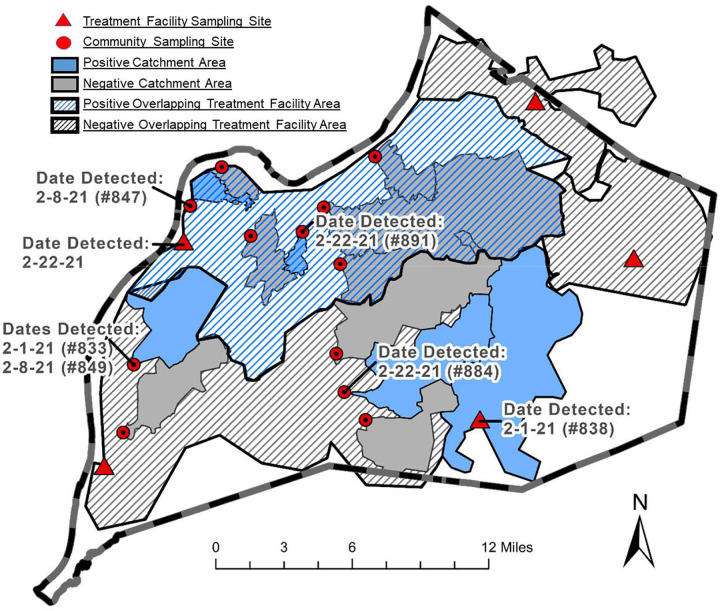
Study sites within Louisville, KY Distribution of the sewershed area, treatment plants and community locations, in Jefferson County with corresponding dates, sampled. SARS-CoV-2 was detected at all sites. Samples that contained at least 50% of the single amino acid mutations for a variant with a nucleotide frequency above a 5 % threshold for individual mutations are flagged for review. This relatively low threshold serves the purpose of identifying geographic (sewershed) areas for heightened public health surveillance. With our current metrics we flagged sites 833, 891, and Treatment plant #2 for potential presence of variant B.1.429.

**Table 1.** Summary of wastewater SARS-CoV-2 samples sequenced in this study, Louisville, KY

Sample ID	Sewershed population	Location	N1 (Ct)	Sequencing BWA Alignment Rate (%)
**833**	35,956	Street line manhole leading to Treatment Plant #3^a^	28	28.02
**Treatment Plant #1**	55,928	Treatment Plant	30	21.09
**847**	10,739	Street line manhole leading to Treatment Plant #2	29	15.08
**849**	35,956	Street line manhole leading to Treatment Plant #3^a^	28	12.61
**884**	46,659	Street line manhole leading to Treatment Plant #3^a^	29	23.98
**891**	8,071	Street line manhole leading to Treatment Plant #2	29	26.03
**Treatment Plant #2**	349,850	Treatment Plant	31	19.96

a Treatment Plant #3 samples had SARS-CoV-2 was detected but were below the threshold for individual mutations for review.

**Table 2.** Summary of B.1.429 specific mutation prevalence by sample

Ref Pos	Gene/ORF	Ref Allele	Alt Allele	Variant Desc	833	Treatment Plant #1	847	849	884	891	Treatment Plant #2
1059	ORF1ab1	C	T	T265I	**0.9309**	**0.8798**	**0.9823**	**0.8906**	**0.9844**	**0.9773**	**0.7382**
12878	ORF1ab1	A	G	14205V	**0.2084**	0	0	0.0015	0	**0.0504**	**0.9968**
14408	ORF1ab2	C	T	P314L	1	1	**0.9975**	1	**0.909**	**0.8757**	**0.8537**
17014	ORF1ab2	G	T	D1183Y	0.049	0.0051	0	0.025	0.0024	0.0026	0.0027
21600	S	G	T	S13I	0	0	0	0	0	0.0025	0
22018	S	G	T	W152C	**0.1287**	0	0	0	0.002	0.0022	0.0016
22917	S	T	G	L452R	**0.1297**	0	0	0	0	0	0
23403	S	A	G	D614G	**0.9972**	1	**0.9969**	1	**0.9969**	**0.9977**	**0.9981**
25563	ORF3a	G	T	Q57H	**0.9893**	**0.6967**	**0.9621**	**0.9987**	**0.8682**	**0.7933**	**0.4046**
28887	N	C	T	T205I	0.0422	0.0426	0	0.0017	0	0	0
