# Supplementary material for: A rapid assessment of wastewater for genomic surveillance of SARS-CoV-2 variants at sewershed scale in Louisville, KY
Source: medRxiv. 2021 Mar 26:2021.03.18.21253604. Preprint. [Version 1] doi: 10.1101/2021.03.18.21253604 (PMC8010757; doi:10.1101/2021.03.18.21253604)
Supplement: 1 [file NIHPP2021.03.18.21253604-supplement-1.pdf]

medRxiv preprint doi: https://doi.org/10.1101/2021.03.18.21253604; this version posted March 26, 2021. The copyright holder for this preprint (which was not certified by peer review) is the author/funder, who has granted medRxiv a license to display the preprint in perpetuity.
It is made available under a CC-BY 4.0 International license .

Supplementary Methods

Wastewater sample Prep

Wastewater samples were collected on February 1, 8 and 22, 2021. In brief, a 24-hour composite raw wastewater sample was collected into a sterile 125ml polyethylene terephthalate bottle. Viral particles where concentrated using PEG precipitation methods. For each sample, 40ml of chilled wastewater was passed through a 70 µm cell strainer and PEG 8000 and (0.5g) NaCl were added to a final concentration of 12.5 mM and 210 mM, respectively. Samples were refrigerated overnight at 4°C and then centrifuged at 16,000 x g for 30mins at 4°C. The pellet was resuspended with 1.1ml TRIzol (Thermo Scientific # 15596018) and transferred to a sterile microfuge tube. The TRIzol sample was then incubated for 5 mins at room temperature and then centrifuged at 12,000 x g for 5 min at 4°C. The sample was then divided into two 500µl samples, one for isolation and one for archiving at -80°C. The sample for isolation had an additional 500µl of TRIzol added and 900µl of 100% Ethanol. Samples were vortexed and the RNA was isolated using a Direct-zol™ 96 MagBead RNA kit (Zymo Research, R2102) with RNA eluted in 100µl of DNAse/RNAse Free Water. RNA cleanup was done using the RNeasy® PowerClean® Pro Cleanup Kit (Qiagen #13995-50) according to the manufacturer's instructions with RNA eluted in 60µl of DNAse/RNAse Free Water. Purified RNA was inspected for yield and quality using a NanoDrop 1000. Number of viral copies in each sample was determined using a probe-based RT-qPCR on a QuantStudio 3 (Applied Biosystems) real-time PCR system using Taq 1-Step Multiplex Master Mix (Thermo Fisher #A28527). The primer and probe sequences are shown in Table 1 with 5 primer/probe sets used for each sample and all samples ran in triplicate. 4µl of sample was used for each 20µl reaction. PCR cycling conditions were 25°C for 2 min, 50°C for 10mins, 95°C for 2 min and 45 cycles of 95°C for 2 sec and 60°C for 30 sec. We generated a standard curve for each primer-probe set used and fit the Ct values to extrapolate copies per mL of wastewater. For this publication we are only reporting on the N1 Ct values generated from this methodology.

cDNA Synthesis

The Superscript® IV First-Strand Synthesis System (Thermo Fisher #18091050) was used to generate cDNA with random hexamer primers. The RT reaction was mixed according to manufacturer’s instructions with a final reaction volume of 20 µl and 5 µl of our template RNA added to the mixture. The reverse transcriptase incubation step was performed with sequential incubation at 23°C for 10 min, 50°C for 30 min, and 80°C for 10 min, according to the manufacturer’s protocol with adjustment of the incubation times recommended by Swift Biosciences SNAP low input protocol.

Library Prep

Libraries were prepared using the Swift Biosciences SNAP low input protocol for SARS-CoV-2 (Swift Bioscience, Ann Arbor, MI, Cat # COSG1V2-96, SN-5X296). 10 µl of cDNA was combined with 20µl of reaction mix and proceeded with multiplex PCR according to protocol. The PCR product was cleaned up using SPRIselect beads (Beckman Coulter, Brea, CA, Cat. No. B23318) at a 1.0X ratio. The purified sample/beads mix was resuspended in 17.4 µl of TE buffer provided in the post-PCR kit. Samples were indexed through PCR with the SNAP Unique Dual Indexing Primers (Swift Bioscience, Ann Arbor, Cat. # SN91096-1-PLATE). The indexing PCR product was

medRxiv preprint doi: https://doi.org/10.1101/2021.03.18.21253604; this version posted March 26, 2021. The copyright holder for this preprint (which was not certified by peer review) is the author/funder, who has granted medRxiv a license to display the preprint in perpetuity. It is made available under a CC-BY 4.0 International license .

further cleaned up and eluted from the beads using a 0.65X PEG NaCl clean-up. The purified libraries were then eluted in 22µL of TE buffer and transferred to fresh tubes and stored at -20°C. For some of the samples (884, 891, and Treatment Plant #2), 1 additional cycle was added to the multiplex PCR and 2 additional cycles were added to the indexing PCR to obtain higher library yields. The library concentration was measured using the Qubit dsDNA HS Assay Kit (Thermo Fisher, Waltham, MA, Q32851). The libraries' size distribution was checked on the Agilent Bioanalyzer using the DNA High Sensitivity Kit (Agilent Technologies, Cat# 5067-4626). Library normalization was performed according to SwiftBio's Normalase 2nM final pool protocol. 5 µl of Normalase I Master Mix were added to each 20µl library eluate for a final pool of 2nM and thoroughly mixed. Samples were placed in the thermocycler to incubate at 30°C for 15 min. 5 µl of each library were pooled, and 1µl of Normalase II Master Mix per library was added and thoroughly mixed. The library pool was placed in the thermocycler to incubate at 37°C for 15 min. 0.2 µl of Reagent X1 per library was added to the pool to inactivate Normalase II at 95°C for 2 min and held at 4°C.

Sequencing

Library pool and PhiX were denatured and diluted following Illumina's directions. Libraries with 1% PhiX spike-in were sequenced at read length 2 x 150 bp using the MiSeq Reagent Kit v2 300 cycle (Illumina, San Diego, CA, Cat# MS-102-2002), or the NextSeq 500/550 Mid Output Kit v2.5 300 Cycles (Illumina, San Diego, CA, Cat# 20024905), targeting 1-5 M reads per library.

Data analysis

Sequencing reads were analyzed using a custom bioinformatics pipeline. Low quality bases were trimmed using Trimmomatic v0.38 (1), and were then aligned to the NC_045512.2 reference genome using bwa mem v 0.7.17-r1188 (2). Single nucleotide variants (SNVs) relative to the reference were detected using bcftools mpileup (3). SNVs occurring in at least 5% of the reads with at least five separate supporting instances were marked for further interrogation. SNVs occurring at locations of interest as they relate to specific SARS-CoV-2 variants (B.1.1.7, B.1.351, B.1.526, P.1, and B.1.429) were reported for all of the samples (Supplementary Methods Tables 2-5).

Table 1. Primer and probe sequences used for RT-qPCR

**Primer Name**	**Sequence**	**Probes**
2019-nCoV_N1-F	5’-GACCCCAAAATCAGCGAAAT-3’	None
2019-nCoV_N1-R	5’-TCTGGTTACTGCCAGTTGAATCTG-3’	None
2019-nCoV_N1-P	5’-**FAM**-ACCCCGCATTACGTTTGGTGGACC-**QSY**-3’	FAM, BHQ-1
RNase P-F	5’-AGATTTGGACCTGCGAGCG-3’	None
RNase P-R	5’-GAGCGGCTGTCTCCACAAGT-3’	None
RNase P-P	5’-**JUN**-TTCTGACCTGAAGGCTCTGCGCG-**QSY**-3’	JUN, BHQ-1
CoV_ORF1ab-F	5’-GTCGTAGTGGTGAGACACTTG-3’	None

medRxiv preprint doi: https://doi.org/10.1101/2021.03.18.21253604; this version posted March 26, 2021. The copyright holder for this preprint (which was not certified by peer review) is the author/funder, who has granted medRxiv a license to display the preprint in perpetuity.
It is made available under a CC-BY 4.0 International license .

CoV_ORF1ab-R	5'-GGCCACCAGCTCCTTTATTA-3'	None
CoV_ORF1ab-P	5'-**FAM**-ATACCAGTGGCTTACCGCAAGGTT-**QSY**-3'	FAM, BHQ-1
PMMoV-F	5'-GAGTGGTTTGACCTTAACGTTTGA-3'	None
PMMoV-R	5'-TTGTCGGTTGCAATGCAAGT-3'	None
PMMoV-P	5'-**VIC**-CCTACCGAAGCAAATG-**QSY**-3'	VIC, BHQ-1
CrAssphage-F	5'-CAGAAGTACAAACTCCTAAAAAACGTAGAG-3'	None
CrAssphage-R	5'-GATGACCAATAAACAAGCCATTAGC-3'	None
CrAssphage-P	5'-**JUN**-AATAACGATTTACGTGATGTAAC-**QSY**-3'	JUN, BHQ-1

Table 2. Summary of B.1.1.7 specific mutation prevalence by sample

Ref Pos	Gene/ORF	Ref Allele	Alt Allele	Variant Desc	833	Treatment Plant #1	847	849	884	891	Treatment Plant #2
3267	ORF1ab1	C	T	T1001I	0	0.0008	0	0	0	0	0
5388	ORF1ab1	C	A	A1708D	0.0015	0	0	0	0.0016	0.0019	0.0016
6954	ORF1ab1	T	C	I2230T	0	0	0	0	0	0	0
11288	ORF1ab1	T	<*>	S3675DEL	0	0	0	0	0	0	0
11289	ORF1ab1	C	<*>	S3675DEL	0	0	0	0	0	0	0
11290	ORF1ab1	T	<*>	S3675DEL	0	0	0	0	0	0	0
11291	ORF1ab1	G	<*>	G3676DEL	0	0	0	0	0	0	0
11292	ORF1ab1	G	<*>	G3676DEL	0	0	0	0	0	0	0
11293	ORF1ab1	T	<*>	G3676DEL	0	0	0	0	0	0	0
11294	ORF1ab1	T	<*>	F3677DEL	0	0	0	0	0	0	0
11295	ORF1ab1	T	<*>	F3677DEL	0	0	0	0	0	0	0
11296	ORF1ab1	T	<*>	F3677DEL	0	0	0	0	0	0	0
21767	S	C	<*>	H69DEL	0	0	0	0	0	0	0
21768	S	A	<*>	H69DEL	0	0	0	0	0	0	0
21769	S	T	<*>	H69DEL	0	0	0	0	0	0	0
21770	S	G	<*>	H70DEL	0	0	0	0	0	0	0
21771	S	T	<*>	H70DEL	0	0	0.0494	0	0	0	0
21772	S	C	<*>	H70DEL	0	0	0	0	0	0	0
21992	S	T	<*>	Y144DEL	0	0	0	0	0	0	0
21993	S	A	<*>	Y144DEL	0	0	0	0	0	0	0
21994	S	T	<*>	Y144DEL	0.0404	0	0	0	0	0	0
23063	S	A	T	N501Y	0	0	0	0	0.0029	0.0038	0.0044
23271	S	C	A	A570D	0	0	0	0	0.0023	0.0021	0.0023
23403	S	A	G	D614G	**0.9972**	**1**	**0.9969**	**1**	**0.9969**	**0.9977**	**0.9981**
23604	S	C	A	P681H	0.0013	**0.0671**	0	0	**0.0645**	0.002	**0.169**
23709	S	C	T	T716I	0.0006	0.0007	0	0.0029	0.0003	0	0.0064
24506	S	T	G	S982A	0	0	0	0	0	0	0
24914	S	G	C	D1118H	0	0	0	0	0	0	0
27972	ORF8	C	<*>	Q27*	0	0	0	0	0	0	0
28048	ORF8	A	G	Y73C	0	0	0	0	0	0	0
28280	N	G	C	D3L	0	0	0	0	0	0	0
28281	N	A	T	D3L	0	0	0	0	0.0009	0.002	0.002
28977	N	C	T	S235F	0.0013	0.0039	0	0.0029	0	0.0013	0

medRxiv preprint doi: https://doi.org/10.1101/2021.03.18.21253604; this version posted March 26, 2021. The copyright holder for this preprint (which was not certified by peer review) is the author/funder, who has granted medRxiv a license to display the preprint in perpetuity.
It is made available under a CC-BY 4.0 International license .

Table 3. Summary of B.1.351 specific mutation prevalence by sample

Ref Pos	Gene/ORF	Ref Allele	Alt Allele	Variant Desc	833	Treatment Plant #1	847	849	884	891	Treatment Plant #2
5230	ORF1ab1	G	T	K1655N	0.0006	0	0	0.0014	0.0023	0.0022	0.0021
22813	S	G	C	K417N	0	0	0	0	0	0	0
23012	S	G	A	E484K	0	0	0	0	0	0	0
23063	S	A	T	N501Y	0	0	0	0	0.0029	0.0038	0.0044
23403	S	A	G	D614G	**0.9972**	**1**	**0.9969**	**1**	**0.9969**	**0.9977**	**0.9981**
23664	S	C	T	A701V	0	0.0018	0.0051	0.0035	0.0009	0	0
26456	E	C	T	P71L	0	0.0069	0	0	0	0	0
28887	N	C	T	T205I	0.0422	0.0426	0	0.0017	0	0	0

Table 4. Summary of B.1.526 specific mutation prevalence by sample

Ref Pos	Gene/ORF	Ref Allele	Alt Allele	Variant Desc	833	Treatment Plant #1	847	849	884	891	Treatment Plant #2
21575	S	C	T	L5F	0	0	0.0051	0	0	0.011	0
21846	S	C	T	T95I	0	0	0	0	0	0	0
22320	S	A	G	D253G	0	0	0	0	0	0	0
23012	S	G	A	E484K	0	0	0	0	0	0	0
23403	S	A	G	D614G	**0.9972**	**1**	**0.9969**	**1**	**0.9969**	**0.9977**	**0.9981**
23664	S	C	T	A701V	0	0.0018	0.0051	0.0035	0.0009	0	0

Table 5. Summary of P.1 specific mutation prevalence by sample

Ref Pos	Gene/ORF	Ref Allele	Alt Allele	Variant Desc	833	Treatment Plant #1	847	849	884	891	Treatment Plant #2
2308	ORF1ab1	T	A	L681L	0	0	0.0008	0	0	0.0012	0.0003
2545	ORF1ab1	T	G	T760T	0	0.0008	0	0	0	0	0
3828	ORF1ab1	C	T	S1188L	0	0	0	0	0	0	0
5648	ORF1ab1	A	C	K1795Q	0	0	0	0.0011	0	0	0
11288	ORF1ab1	T	<*>	S3675DEL	0	0	0	0	0	0	0
11289	ORF1ab1	C	<*>	S3675DEL	0	0	0	0	0	0	0
11290	ORF1ab1	T	<*>	S3675DEL	0	0	0	0	0	0	0
11291	ORF1ab1	G	<*>	G3676DEL	0	0	0	0	0	0	0
11292	ORF1ab1	G	<*>	G3676DEL	0	0	0	0	0	0	0
11293	ORF1ab1	T	<*>	G3676DEL	0	0	0	0	0	0	0
11294	ORF1ab1	T	<*>	F3677DEL	0	0	0	0	0	0	0
11295	ORF1ab1	T	<*>	F3677DEL	0	0	0	0	0	0	0
11296	ORF1ab1	T	<*>	F3677DEL	0	0	0	0	0	0	0
21614	S	C	T	L18F	0	0	0	0	0	0	**0.338**
21621	S	C	A	T20N	0	0	0	0	0.0006	0.0026	0
21638	S	C	T	P26S	0	0	0	0	0	0	0
21974	S	G	T	D138Y	0	0	0	0	0.0026	0.0025	0.0017
22132	S	G	T	R190S	0	0	0	0	0.0012	0.0021	0.0028
22812	S	A	C	K417T	0	0	0	0	0	0	0
23012	S	G	A	E484K	0	0	0	0	0	0	0
23063	S	A	T	N501Y	0	0	0	0	0.0029	0.0038	0.0044
23403	S	A	G	D614G	**0.9972**	**1**	**0.9969**	**1**	**0.9969**	**0.9977**	**0.9981**
23525	S	C	T	H655Y	0	0	0	0	0	0	0
24642	S	C	T	T1027I	0	0	0	0	0	0	0
25912	ORF3a	G	T	G174C	0	0	0	0	0.0015	0.0009	0.0013
28167	ORF8	G	A	E92K	0	0.0006	0	0	0	0	0.0016
28512	N	C	G	P80R	0	0	0	0	0	0	0

medRxiv preprint doi: https://doi.org/10.1101/2021.03.18.21253604; this version posted March 26, 2021. The copyright holder for this preprint (which was not certified by peer review) is the author/funder, who has granted medRxiv a license to display the preprint in perpetuity.
It is made available under a CC-BY 4.0 International license .

References:

1. Bolger AM, Lohse M, & Usadel B (2014) Trimmomatic: a flexible trimmer for Illumina sequence data. *Bioinformatics* 30(15):2114-2120.
2. Heng L (2013) Aligning sequence reads, clone sequences and assembly contigs with BWA-MEM. *arXiv*:1303.3997.
3. Li H & Durbin R (2009) Fast and accurate short read alignment with Burrows-Wheeler transform. *Bioinformatics* 25(14):1754-1760.
